# Translational approach to assess brain injury after cardiac arrest in preclinical models: a narrative review

**DOI:** 10.1186/s40635-024-00710-y

**Published:** 2025-01-14

**Authors:** Francesca Motta, Marianna Cerrato, Daria De Giorgio, Alice Salimbeni, Giulia Merigo, Aurora Magliocca, Carlo Perego, Elisa R. Zanier, Giuseppe Ristagno, Francesca Fumagalli

**Affiliations:** 1 Department of Acute Brain and Cardiovascular Injury Istituto di Ricerche Farmacologiche Mario Negri IRCCS, Milan, Italy; 2 Department of Biomedical Sciences for Health, University of Milan, Milan, Italy; 3 Department of Pathophysiology and Transplants, University of Milan, Milan, Italy; 4 Department of Anesthesia, Critical Care and Emergency, Fondazione IRCCS Ca’ Granda - Ospedale Maggiore Policlinico, Milan, Italy

## Introduction

Post-cardiac arrest brain injury (PCABI) is the main cause of death and disability in cardiac arrest (CA) patients [1]. Our understanding of the processes linking the cellular and molecular mechanisms that mediate brain structural and functional damage after CA is still incomplete, partly because of the paucity of relevant preclinical studies.

Increasing evidence suggests that, despite the many positive results from experimental studies, several neuroprotective drugs have failed to demonstrate clinical benefits in humans after CA [2]. This discrepancy may be attributed to biases or misinterpretation of preclinical results, such as the use of cerebral ischemia models without CA, unclear reporting of random assignment, lack of blinding and power analysis, poor reproducibility and insufficiently detailed study protocols [3]. In addition, there is often a failure to rigorously adhere to ARRIVE guidelines [4]. To improve the translatability of experimental results, preclinical study designs should more closely align with clinical settings. Indeed, many preclinical findings are difficult to translate into clinical practice, partly due to differences in injury models and functional assessment methods [3].

In PCABI animal studies, neurological damage and outcome are typically assessed using mainly neuropathology and animal-specific neurological deficit scores [3], with the addition of blood biomarkers of brain injury and neuroimaging techniques in some cases. In clinical practice, neuroprognostication after cardiac arrest relies on a multimodal approach incorporating clinical examination, circulating biomarkers, neurophysiology, and imaging to enhance diagnostic accuracy [5]. However, experimental studies rarely employ a multimodal approach or focus on evaluating whether individual methods correlate strongly with functional outcomes, contributing to the frequent failure in translating preclinical findings to clinical settings. To address this gap, it is crucial that preclinical research adopts methodologies similar to those used clinically. Drawing from clinical practice, preclinical studies should emphasize combining multiple techniques to provide a comprehensive assessment of brain injury evolution and recovery.

This narrative review aims to provide a comprehensive overview of the translational methods employed in preclinical research for evaluating brain injury and neurological recovery after CA. It seeks to analyze how different methods such as histological analysis, blood biomarkers, neurophysiology and neuroimaging techniques are employed in preclinical studies and will highlight the strengths and limitations of these methods in correlating with functional outcomes. The primary objective is to identify methodological approaches that more accurately reflect functional outcomes in the animal models. In addition, the review seeks to propose a multimodal approach that combines different techniques in animal’s studies, with the goal of bridging the gap between preclinical findings and clinical outcomes.

## Materials and methods

### Search strategy

**Fig. 1:**
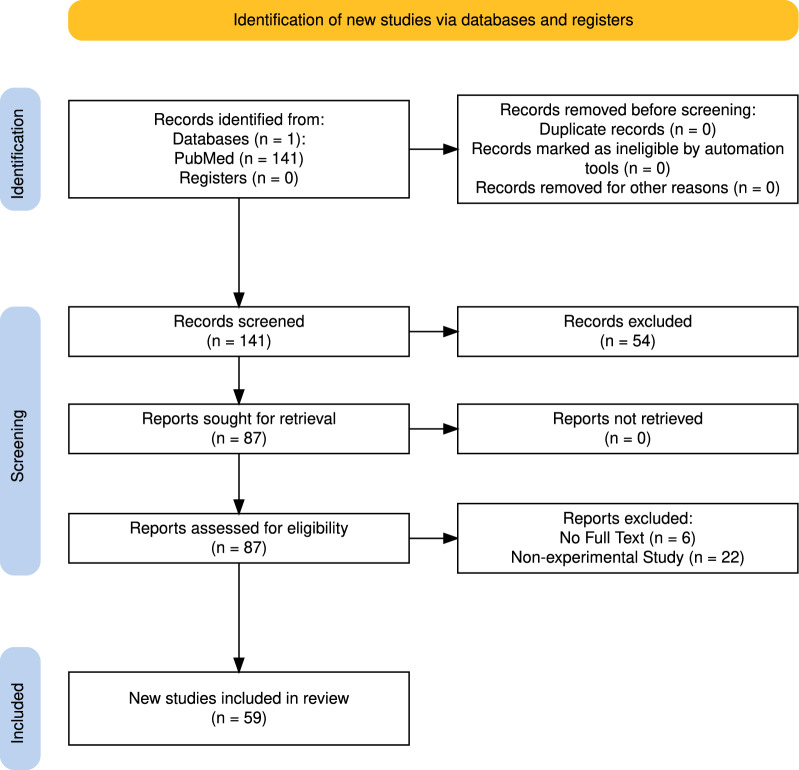
PRISMA flow diagram for literature search and study selection process

This narrative review was conducted in accordance with the Preferred Reporting Items for Systematic Reviews and Meta-analysis (PRISMA, Fig. 1) [6, 7]. The PubMed database was selected as the primary source for data retrieval. We identified studies focusing on CA, brain injury and neurological outcomes in animal models published between 2021 and 2024. The search combined both keywords, Title/Abstract (Tiab) and MeSH (Medical Subject Headings) terms to ensure comprehensive coverage of relevant literature.

((cardiac arrest [Tiab]) OR (cardiopulmonary resuscitation [Tiab]) OR (cardiopolmunary resuscitation [MeSH]) OR (heart arrest [MeSH])) AND ((brain injury [Tiab]) OR (neurological outcome [Tiab]) OR (neurological recovery [Tiab]) OR (neurological dysfunction [Tiab]) OR (brain dysfunction [Tiab]) OR (neuroprotection [MeSH]) OR (brain injuries [MeSH]) OR (recovery of function [MeSH])) AND animal [Filter] AND (2021:2024[pdat]).

Articles eligible for inclusion in this narrative review were independently assessed for quality by two authors (FM and MC) and differences in scoring were resolved through discussion with the corresponding author (FF). The selection criteria further refine the search 141 results and after application of selection criteria, we identified 59 eligible studies.

### Data collection

From all eligible articles, the following information were recorded: PMID number, DOI reference, name of the first author, publication year, title, aim, CA model, animal model, description of neurological function evaluation, histopathology, imaging, electrophysiology, circulating biomarkers, and main findings.

### Selection criteria

The selection of studies was guided by specific criteria to ensure a comprehensive evaluation of brain injury and recovery following CA in animal models. The primary inclusion criteria was the presence of functional outcome tests. Only studies that included assessments of functional outcomes, tests that measure behavioral, cognitive, or neurological recovery, were considered. Another inclusion criterion was the evaluation of brain injury. Studies needed to include at least one of the following methods for assessing brain injury:

- **Histological evaluation**: this involves the microscopic examination of brain tissue to assess structural damage at the cellular level.
- **In vivo neuro imaging techniques**: methods such as magnetic resonance imaging (MRI) or computed tomography (CT) scans are used to visualize brain injury or recovery, offering a non-invasive way to track changes in brain structure and function over time.
- **Electroencephalography (EEG):** monitoring of brain electrical activity to assess the severity of brain dysfunction and track neurological recovery over time.
- **Circulating biomarkers**: measurement of specific protein biomarkers, such as S100 calcium‐binding protein B (S100B), neuro-specific enolase (NSE), neurofilament light (Nfl) and lactate, in the bloodstream. These biomarkers reflect multi-organ failure and neuronal damage and are valuable for assessing the severity and progression of brain injury after CA.

By adhering to these inclusion criteria, this review aims to highlight studies that not only examine the extent of brain damage but also provide methodological approaches that more accurately reflect functional outcomes.

## Results

The search identified 59 articles (Tables 1, 2, 3, 4, 5 and 6). Of these, 39 were models of asphyxia-induced CA (Tables 1 and 2), 6 were model of asystolia-induced CA (Table 3) and 15 were models of ventricular fibrillation-induced CA (Tables 4, 5 and 6). The search was not limited to a single species, but it encompasses a diverse range of species: seven articles used mice model, one used a rabbit model, 43 employed rats and eight works pigs.

**Table 1:** Asphyxia-induced CA in rats

Ref	Study	No flow/Low flow time	ROSC rate	Survival	Survival Rate	Neurofunction	NDS score	Histology	Imaging	EEG	Biomarker	Score
[38]	Xia et al	5 min + 5 min	n.a	24 h and 3d	n.a	NDS (0, death; 80, normal)	≈ 40	Nissl, Iba1 and GFAP	/	/	/	
[24]	Chen et al	10 min + 5 min	n.a	24 h and 8d	≈80%; ≈45%	NDS (0, death;10, normal); Morris Water Maze	≈ 2.5	/	/	/	NSE and S100B	
[25]	Xu et al	5 min + 40 s	n.a	24 h and 14d	n.a	NDS (0, death; 80, normal); Morris Water Maze	≈ 40; ≈ 63	H&E, TUNEL and NeuN	/	/	NSE and S100B	
[39]	Chang et al	7 min + 30 min	n.a	4 h	n.a	NDS (0, death; 500, normal)	≈ 45	GFAP and S100B	/	/	Lactate	
[40]	Li et al	7 min + 3 min	100%	4d	30%	NDS (0, normal; 500, death)	[429;500]	H&E and Fluoro-Jade	/	/	S100B	
[41]	Dai et al	8 min + 10 min	60% and 66.7%	3d	33.8%	NDS (0, death; 80, normal)	≈ 68	H&E and TUNEL	/	/	NSE and S100B and Lactate	
[42]	Hu et al	8 min + n.a	42%	24 h	41%	NDS (0, normal; 500, death); OPC (1, normal; 5, death)	[150;500]	Nissl, Cyt-c and SIRT3	/	/	/	
[43]	Zhan et al	8 min + n.a	n.a	7d	n.a	NDS (0, death; 80, normal)	≈ 72	H&E, Nissl and TUNEL	/	/	/	
[44]	Zhou et al	10 min + 5 min	n.a	7d	40%	NDS (0, death; 80, normal)	≈ 60	Nissl and TUNEL	/	/	/	
[45]	Tang et al	8 min + n.a	n.a	24 h	n.a	NDS (0, death; 80, normal)	≈ 65	H&E, NLRP3 and Cleaved-caspase-1	/	/	NSE and S100B	
[46]	Zhang et al	10 min + n.a	n.a	24 h and 7d	n.a	NDS (0, death; 80, normal); Morris Water Maze	≈ 60	TUNEL, H&E and Nissl	/	/	/	
[47]	Choudhary et al	20 min + 30 min; 12 min + n.a	n.a	3d	28.6%	1. NDS (0, death; 500, normal); 2. NDS (0, death; 80, normal)	≈ 250; ≈ 42	Nissl, NeuN, TUNEL and GFAP	/	/	/	
[48]	Hayashida et al	10 min + n.a	n.a	3d	58%	NFS (0, death; 500, normal)	≈190	/	/	/	Lactate	
[26]	Oghifobibi et al	5 min + < 1.5 min	n.a	10d	n.a	Balance Beam Test and Morris Water Maze	n.a	Nissl	/	/	/	
[49]	Wang et al	6 min + 10 min	93%	24 h	≈80%	NFS (0, death; 12, normal)	≈9	TUNEL	/	/	NSE and S100B and Lactate	
[27]	Xia et al	10 min + < 2 min	n.a	3d and 7d	85.4%; 68.6%	NDS (0, normal; 18, death); Morris water maze	≈8	H&E, Nissl and TUNEL	/	/	/	
[28]	He et al	10 min + n.a	81%	7d	n.a	NDS (0, death; 80, normal); Morris Water Maze	≈70	Nissl, GFAP, NeuN, MAP2, Iba1 and CD68	/	/	/	
[50]	Jackson et al	8–9 min + n.a	n.a	7d	n.a	NDS (0, normal; 500, death); OPC (1, normal; 5, death)	≈ 25; ≈1.2	H&E	/	/	/	
[51]	Guo et al	7 min + n.a	71%	24 h	100%	NDS (0, death; 80, normal)	≈ 67	/	MRI	/	/	
[32]	Tan et al	8 min + 6 min	n.a	24 h	45%	NDS (0, death; 80, normal); Y-Maze	≈ 45	H&E, Nissl and TUNEL	/	/	Lactate	
[52]	Liu et al	8 min + n.a	n.a	3d and 7d	50%	NDS (0, death; 80, normal)	≈ 40	Nissl	/	/	/	
[33]	Lee et al	6 min + n.a	n.a	3d and 7d	≈80%	Y-Maze	n.a	H&E and Fluoro-Jade	/	/	/	
[11]	Shoaib et al	10 min + 10 min	n.a	3d	40%	NDS (0, death; 500, normal)	≈ 250	TUNEL, Nissl and GFAP	/	EEG	Lactate	
[23]	Sun et al	10 min + 5 min	n.a	3d	n.a	Object recognition test (ORT) and Object location test (OLT)	n.a	Iba1, NLP3, Caspase 1 and GSDMD	/	/	/	
[53]	Zhang et al	12 min + 1.20 min	68.75%	3d and 7d	n.a	NDS (0, death; 80, normal)	≈ 73	DCX, RBM3, NeuN and TUNEL	/	/	/	
[34]	Nishikimi et al	10 min + 1 min	n.a	3d	n.a	Neuronal reflex test (0, death; 100, normal); Motor coordination test (0, death; 100, normal)	≈ 40	GFAP, NeuN, Iba1 and anti-β-tubulin III	/	/	/	
[54]	Wu et al	9.5 min + 3 min	90%	3d	60%	NDS (0, death; 12, normal)	≈ 40	H&E	/	/	/	
[55]	Liu et al	8 min + 10 min	n.a	3d	100%	NDS (0, normal; 500, death)	133.6	H&E, and NSE	MRI–DWI and DCE–MRI	/	/	
[14]	Wang et al	7 min + n.a	n.a	4d	35% Female; 15% male	NDS (0, normal; 500, death)	≈ 500	/	/	EEG	/	
[56]	Shao et al	5 min + 5 min	n.a	21d	n.a	NDS (0, death; 80, normal); Balance Beam Test	n.a	Nissl, Iba1, NeuN, GFAP	/	/	/	
[57]	Yang et al	6 min + 4 min	n.a	3d	100%	NDS (0, normal; higher score, higher deficit)	165	TUNEL	/	/	/	
[29]	Liu et al	8 min + 5 min	n.a	7d	n.a	Morris Water Maze; Balance Beam; Prehensile traction test	n.a	H&E, TUNEL, Iba1 and NeuN	/	/	/	
[58]	Zhang et al	8 min + 5 min	n.a	10d	n.a	NDS (0, death; 500, normal)	70	Nissl	/	/	Lactate	
[12]	Wang et al	8 min + 2 min	n.a	3d	n.a	NDS (0, death; 80, normal)	≈ 60	Nissl	/	EEG	/	
[59]	Huang et al	5 min + n.a	n.a	3d	n.a	NDS (0, death; 80, normal)	64.42	Nissl, TUNEL and NeuN	/	/	/	
[13]	Dai et al	8 min + n.a	n.a	4d	67%	NDS (0, normal; 500, death)	≈100	/	/	EEG	/	

*ROSC* return of spontaneous circulation, *NDS* Neurological Deficit Score, *NFS* Neurological Function Score, *H&E* Hematoxylin and Eosin staining, *EEG* electroencephalography exam, *MRI–DWI* diffusion-weighted imaging, *MRI–DTI* diffusion tensor imaging, green squares correspond to methods employed: one green square corresponds to one method, two green squares correspond to two methods, three green squares correspond to three methods. The ROSC, survival rate and NDS score refer to the CA control group

**Table 2:** Asphyxia-induced CA in mice

Ref	Study	No flow/Low flow time	ROSC rate	Survival	Survival Rate	Neurofunction	NDS score	Histology	Imaging	EEG	Biomarker	Score
[22]	Duan et al	10 min + < 2 min	100%	3d	57%	Open-field test; Rotarod test	n.a	Fluoro-Jade	/	/	/	

*ROSC* return of spontaneous circulation, *NDS* Neurological Deficit Score, *NFS* Neurological Function Score, *H&E* Hematoxylin and Eosin staining, *EEG* electroencephalography exam, *MRI–DWI* diffusion-weighted imaging, *MRI–DTI* diffusion tensor imaging, green squares correspond to methods employed: one green square corresponds to one method. The ROSC, survival rate and NDS score refer to the CA control group

**Table 3:** Asystolia-induced CA in mice

Ref	Study	No flow/Low flow time	ROSC rate	Survival	Survival Rate	Neurofunction	NDS score	Histology	Imaging	EEG	Biomarker	Score
[9]	Magliocca et al	8 min + 1,5 min	75%	7d	37.5%	NFS (0, death; 10, normal); Spontaneous locomotor activity	7	Iba1 and GFAP	MRI–DWI and DTI	/	NFL	
[20]	Peng et al	8 min + ≤ 3 min	n.a	3d	50%	NFS (0, death; 12, normal); Fear conditioning test; Novel Object Recognition; Open Field Test	5	Fluoro-Jade	/	/	/	
[21]	Chen et al	10 min + 0–3,5 min	n.a	7d	35%	NFS (0, death; normal, 10); Rotarod test; Open Field Test; Morris Water Maze test	7	Fluoro-Jade, TUNEL, NeuN, Iba1 and GFAP	MRI–DWI	/	/	
[60]	Li et al	6 min + 2,5 min	n.a	7d	38%	NDS (0, death; 80, normal)	≈25	H&E and TUNEL	/	/	/	
[31]	Ousta et al	12 min + ≤ 5 min	61%	3d	39%	NDS (0, death; 12, normal); Tape Removal Test	8.1	Fluoro-Jade and Iba1	/	/	/	
[10]	Zhang et al	12 min + ≤ 5 min	n.a	3d	64%	NDS (0, death; 12, normal)	≈7	/	PET/CT	/	/	

*ROSC* return of spontaneous circulation, *NDS* Neurological Deficit Score, *NFS* Neurological Function Score, *H&E* Hematoxylin and Eosin staining, *EEG* electroencephalography exam; *MRI–DWI* diffusion-weighted imaging, *MRI–DTI* diffusion tensor imaging, green squares correspond to methods employed: one green square corresponds to one method, two green squares correspond to two methods, three green squares correspond to three methods. The ROSC, survival rate and NDS score refer to the CA control group

**Table 4:** Ventricular fibrillation-induced CA in rats

Ref	Study	No flow/Low flow time	ROSC rate	Survival	Survival Rate	Neurofunction	NDS Score	Histology	Imaging	EEG	Biomarker	Score
[8]	Perego et al	8 min + 8 min	92%	14 d	67%	NDS (0, death; 500, normal); Open Field Test; Tape Removal; Spontaneous Locomotor Activity	450	Nissl, TUNEL, Fluoro-Jade, Iba1and GFAP	MRI–DWI and DTI	/	NfL	
[40]	Li et al	7 min + 3,5 min	100%	4 d	37,5%	NDS (0, normal; 500, death)	[100; 500]	H&E and Fluoro-Jade	/	/	S100B	
[19]	Zhang et al	7 min + 5 min	n.a	7 d	n.a	NDS; Open Field Test; Rotarod Test	≈3	TUNEL, NeuN, Iba1 and β III Tubulin	/	/	/	
[30]	Cai et al	4 min + < 2 min	87%	7 d	40%	Tape removal test	n.a	MMP9	/	/	/	
[61]	Ye et al	8 min + 6 min	87%	24 h	n.a	NDS (0, death; 80, normal)	≈40	H&E and TUNEL	/	/	/	
[62]	Yuan & Zhang	7 min + < 3 min	n.a	24 h	67%	NDS (0, death; 80, normal)	66.2	Nissl and TUNEL	/	/	/	
[63]	Wang et al	7 min + 1,5 min	n.a	24 h	56,25%	NDS (0, death; 80, normal)	70	H&E and Nissl, TNF-a	/	/	/	
[64]	Tsai et al	5 min + 1 min	64%	3 d	60%	NDS (0, death; 12, normal)	20% good outcome	H&E	/	/	/	

*ROSC* return of spontaneous circulation, *NDS* Neurological Deficit Score, *NFS* Neurological Function Score, *H&E* Hematoxylin and Eosin staining, *EEG* electroencephalography exam, *MRI–DWI* diffusion-weighted imaging, *MRI–DTI* diffusion tensor imaging, green squares correspond to methods employed: one green square corresponds to one method, two green squares correspond to two methods, three green squares correspond to three methods. The ROSC, survival rate and NDS score refer to the CA control group

**Table 5:** Ventricular fibrillation-induced CA in pigs

Ref	Study	No flow/Low flow time	ROSC rate	Survival	Survival rate	Neurofunction	NDS score	Histology	Imaging	EEG	Biomarker	Score
[65]	Motta et al	12 min + 5 min	75%	4 d	n.a	OPC (1, normal; 5, death)	50% good outcome	H&E, Fluoro-Jade, Iba1 and GFAP	/	/	/	
[66]	Shen et al	10 min + 5 min	67%	24 h	100%	NDS (0, normal; 400, death)	≈150	/	/	/	NSE and S100B	
[67]	Wider et al	8 min + 2–5 min	82%	4 d	n.a	NDS (0, normal; 150, death)	≈40	Nissl	/	/	/	
[68]	Vammen et al	9 min + 2–20 min	83%	9 d	78%	NDS (0, normal; 300, death); OPC (1, normal; 5 death); Visual–spatial test; human approach test	NDS: 0 OPC: 78% good outcome	/	/	/	NSE and lactate	
[69]	Pooth et al	20 min + 60 min	100%	7 d	75%	NDS (< 50, normal)	0	/	/	/	NSE	
[70]	Wang et al	8 min + 2–20 min	n.a	24 h	n.a	NDS (0, normal; 400, death)	≈200	/	/	/	Lactate and NSE and S100b	
[71]	Diao et al	8 min + 8 min	69%	24 h	100%	NDS (0, normal; 400, death)	209	Cleaved Caspase-1 and GSDMD	/	/	NSE	
[72]	Shen	8 min + 5 min	86%	24 h	86%	NDS (0, normal; 400, death)	≈200	TUNEL, Caspase 3 and Caspase 8	/	/	NSE and S100B	

*ROSC* return of spontaneous circulation, *NDS* Neurological Deficit Score, *OPC* Overall Performance Category, *H&E* Hematoxylin and Eosin staining; green squares correspond to methods employed: one green square corresponds to one method, two green squares correspond to two methods, and three green squares correspond to three methods. The ROSC, survival rate and NDS score refer to the CA control group

**Table 6:** Ventricular fibrillation-induced CA in rabbits

Ref	Study	No flow/Low flow time	ROSC rate	Survival	Survival rate	Neurofunction	NDS score	Histology	Imaging	EEG	Biomarker	Score
[73]	Boissady et al	8 min + 5 min	n.a	3 d	20%	NDS (0%, normal; 100%, death)	100%	Fluoro-Jade	/	/	Lactate	

*NDS* Neurological Deficit Score, *ROSC* return of spontaneous circulation, green squares correspond to methods employed: one green square corresponds to one method, two green squares correspond to two methods, and three green squares correspond to three methods. The ROSC, survival rate and NDS score refer to the CA control group

This analysis showed that histology was the most commonly used technique, with 82% of studies employing histological or immunohistochemical evaluations to assess brain injury. In contrast, circulating biomarkers, imaging, and EEG were used less frequently, appearing in 33%, 10%, and 7%, of animal studies, respectively.

Survival rates were tracked in most studies at various timepoints, including 24 h and 72 h post-CA. A subset of studies extended the monitoring period to 14 days, which is critical for understanding long-term outcomes.

Neurological functional outcome was measured by general neurological behavior test using clinical deficit scores with specific grids for each species. In other 23 studies, animals underwent behavioral testing, to assess distinct cognitive behavior, such as Open Field, Y-Maze, Novel Object recognition, Morris Water Maze, Rotarod and Tape Removal tests.

**Fig. 2:**
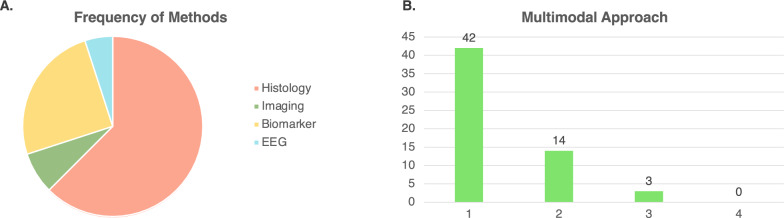
**A**. Frequency of methods employed between Histology, Imaging, Biomarkers and EEG; **B**. Frequency of combination of method. Score 1, only one method employed; score 2, two methods employed; score 3, three methods employed

Forty-nine articles employed histological examination to measure the severity of PCABI. Common markers included Nissl, Iba1, GFAP, and Tunel, which were used to analyze neuronal damage, microglial and astrocytes activation and apoptosis (Fig. 2A).

Six studies employed advanced neuroimaging techniques. MRI–diffusion-weighted imaging (MRI–DWI) and MRI–diffusion tensor imaging (MRI–DTI) were used to evaluate structural brain damage in five studies. In two studies [8, 9], MRI was used in combination with other assessments, such as neurological function or biomarker measurements. This multimodal approach offers a more comprehensive understanding of both functional and structural changes in the brain. One study employed combined CT scans as a reference for precise anatomical localization of glucose uptake in different brain regions [10]. In general, this analysis indicates a moderate reliance on advanced imaging techniques, primarily MRI, for detecting structural damage (Fig. 2A).

Only 4 studies used EEG approach to evaluate neurophysiology [11–14], indeed real-time functional assessment of brain injury remains underexplored in these preclinical studies (Fig. 2A).

Our analysis revealed that in 20 papers circulating biomarkers were evaluated, with NSE and S100B, being the most used. Lactate was analyzed in 8 different studies, as marker of metabolic distress and brain hypoxia (Fig. 2A). Meanwhile, NfL was measured in only two studies.

To determine whether a multimodal approach was employed in evaluating PCABI we also examined how many experimental studies utilized more than one method. Among the 59 articles reviewed, none fully adopted a multimodal approach. Only two studies used three methods to evaluate PCABI in mouse and rat models, specifically combining histological analysis, MRI and NfL levels [8, 9]. One study, investigated PCABI employing histological analysis, EEG evaluation and lactate levels after CA [11]. In contrast, the majority of the studies employed only one or two techniques, with 42 using a single approach and 14 using two (Fig. 2B).

## Discussion

Our analysis reveals significant discrepancies between preclinical study designs and clinical practice, underscoring the urgent need for more clinically relevant and multimodal approaches to enhance the translational potential of experimental findings. Specifically, our study shows that most of preclinical studies on CA were not designed based on a clinically derived approach, thus limiting the translatability of results.

### The need for neuroprotective treatments

There is an urgent need to identify novel neuroprotective treatments after CA. Over the past few decades, many treatments have been tested in the preclinical setting. Remarkably, the majority of them showed promising results in animal studies but failed to replicate the same success in large clinical trials. One reason for this translational failure is the discrepancy between the methods used in preclinical research and those applied in clinical practice.

In clinical neuroprognostication—the prediction of neurological recovery—it is recommended to use a multimodal approach as no single test has sufficient specificity to avoid false positives[2]. Embracing a multimodal methodology in animal research, could increase the translatability of findings, mirroring clinical practice, where combining multiple assessment tools improves the prediction of neurological outcomes.

### Post-resuscitation care and neuroprognostication

The post-resuscitation care in years has gained increasing importance. In 2010 guidelines, the post-resuscitation care was a paragraph incorporated in Advance Life Support section [15]. In 2015, the ERC and ESICM published guidelines specifically focused on the post-resuscitation care section, emphasizing the importance of high-quality post-resuscitation care and highlighting how this is a crucial factor in the chain of survival. Moreover, in 2015, it has been introduced for the first time the concept of the multimodal approach for neuroprognostication [16].

Two-thirds of deaths occurring in OHCA patients are due to PCABI, with the majority resulting from the active withdrawal of life-sustaining treatment (WLST) based on neuroprognostication. Indeed, it was important to minimize the risk of falsely poor prediction [16]. ERC guidelines 2015, suggested for the first time a multimodal neuroprognostication strategy, so a combination of distinct parameters that can increase the sensitivity to predict a poor outcome in patients. Specifically, this prognostication strategy algorithm was composed by one or both of no pupillary and corneal reflexes and bilaterally absent N20 SSEP wave [16]. In addition, they recommended that in none of those signs were present, it can be evaluated also a group of less accurate predictors: the presence of early status myoclonus, high values of NSE at 48–72 h after Return of Spontaneous Circulation (ROSC), an unreactive malignant EEG pattern and the presence of anoxic injury evaluated by brain CT or brain MRI scans [16]. ERC and ESICM proposed this algorithm based on evidence that none of these predictors singularly predicted poor outcome with 100% of accuracy, but combined together, then, they can increase safety and sensitivity to avoid falsely pessimistic prediction.

In the latest 2021 guidelines the concept of neuroprognostication has been revisited and improved: since 2015 there has been many studies regarding prognostication, which validated and confirmed the reliability of the algorithm presented in the last guidelines of 2015. Therefore, based on these data, they simplified the two-stage strategy algorithm so that poor outcome is considered likely when two or more listed predictors are present: no pupillary or corneal reflex at ≥ 72 h, bilaterally absent N20 SSEP wave at ≥ 24 h, NSE > 60 μg/L at 48 h and/or 72 h, presence of status myoclonus ≤ 72 h and a diffuse and extensive anoxic brain injury on CT or MRI scans [2].

### The role of histological analysis

Histological analysis is an essential component of preclinical research, providing critical insights into the biological mechanisms underlying injury evolution following CA. Although the findings from histological studies are not always translatable to clinical settings, they are vital for understanding the pathophysiological changes that occur after brain injury. Histological and immunohistochemical methods remain essential tools for evaluating the severity of PCABI. Techniques such as hematoxylin and eosin (H&E), Nissl and Fluoro-Jade staining allow researchers to examine neuronal death and neurodegeneration. [17]. Immunohistochemistry is particularly valuable for investigating the inflammatory response following CA [17], focusing on key cellular populations like microglia and astrocytes, which mediate the brain's immune response and hold a pivot role in contributing to long term outcome. [1, 5]. These glial cells are highly active in the injured brain and play a pivotal role as immune cells that mediate inflammation. Their behavior and activation states, as revealed through immunohistochemistry, provide important clues about the extent and nature of the brain's response to injury after CA [1, 5]. Susceptibility to ischemia reperfusion injury due to CA, varies significantly depending on the neuronal subtype and region, with area more susceptible than others: the neocortex, the hippocampus, basal ganglia, cerebellum and thalamus [5].

However, it is essential to recognize that histological analysis cannot be obtained at the bedside and not always directly correlate with functional outcomes, which can limit its applicability in clinical contexts. Therefore, integrating histological analysis with other assessments, such as neuroimaging, biomarkers, and functional outcome measures, is crucial for a more comprehensive understanding of brain injury and recovery in conjunction with behavioral assessments.

### Multimodal approaches in preclinical studies

Inspired by the multimodal approach used in clinical practice for neuroprognostication, we propose applying a similar strategy in preclinical research to improve the assessment of brain injury and functional recovery after CA.

### Functional outcome

Given the poor prognosis of cardiopulmonary resuscitation (CPR) with regard to both survival and neurological outcome, functional evaluation constitute one of the primary measures of outcome [18].

In preclinical research on CA, neurological deficit tests play a critical role in assessing the extent of brain injury and functional recovery in animal models. These tests can be broadly categorized into two groups: those evaluating general neurological behavior and those focused on cognitive and behavioral assessments.

#### General neurological behavior

General neurological behavior tests provide a basic overview of the animal’s neurological status post-CA. These tests are essential for identifying the immediate and overt effects of brain injury. Specifically, distinct aspects of animal’s behavior can be assessed using clinical deficit scores with specific grids for each species.

They are based on the clinical evaluation of consciousness, behavior, breathing, reflexes, coordination, motor and sensory activity and seizure. The consciousness and general behavior, is asses by observing any changes in movement or responsiveness of the animal. The brainstem performance, is the evaluation of reflexes controlled by brainstem, i.e., pupillary, responses and breathing. The coordination, is assessed by testing the motor coordination through balance observations. Furthermore, the corneal reflex is evaluated by blink response to corneal stimulation and it indicates the cranial nerve function. The motor and sensory activity is the assessment of animal’s voluntary movements and response to sensory stimuli. Finally, is often present the study of seizure activity, so monitoring the occurrence of seizure or convulsions post-CA.

These analyses provide insight into the severity of neurological damage and recovery following CA. However, they primarily focus on broad neurological outcomes and may not capture the full spectrum of cognitive or behavioral impairments.

#### Cognitive and behavioral assessments

The second group offers a more specific evaluation of specific brain functions. These tests explore aspects like memory, anxiety, exploratory behavior, and learning, critical in understanding the deeper impact of CA on brain function. The key tests in this category include:

- Open field test: used to assess anxiety levels and exploratory behavior by measuring the animal's movement and interaction with a novel environment [8, 19–22];
- Novel object recognition test: evaluates recognition memory, based on the animal's ability to differentiate between familiar and new objects [20, 23];
- Morris water maze: a well-established test for assessing spatial learning and memory, where animals must navigate to a hidden platform in a pool of water [19, 21, 24–29];
- Tape removal test: evaluating sensorimotor function by placing adhesive tape on the forepaw and recording the time it takes for the animal to detect and remove it, reflecting sensory and motor coordination [8, 30, 31];
- Y-maze: Used to assess working memory by analyzing spontaneous alternation behavior as the animal explores different arms of the maze [32, 33];
- Motor activity test: assesses general locomotor activity by tracking the animal’s movement, measuring distinct parameters like distance traveled, speed, time spent moving versus resting and spontaneous motor activity during light and dark phase of day [8, 9, 19, 21, 22, 34].

These cognitive and behavioral tests are particularly valuable for examining long-term brain function, providing a more detailed understanding of how CA impacts learning, memory, and emotional regulation.

### Biomarkers

Multi-organ failure and neuronal damage can be measured in the serum or plasma after CA as biomarkers. Major advantages of blood biomarkers are that they are easy to obtain and offer a quantitative and easily interpreted measure of the extent of brain injury [1].

#### Neuron-specific enolase

NSE is a neuronal glycolytic enzyme that is abundant in the neurons of brain gray matter and involved in axonal transport [35]. In healthy individuals, serum levels of NSE remain low. In contrast, upon damage to neuronal tissue, such as anoxic brain injury, NSE serum concentration increases and consequently acts as a biomarker for brain damage. European Resuscitation Council (ERC) guidelines 2021 indicates that increasing values between 24 and 48 h or 72 h in combination with high values at 48 and 72 h indicate a poor prognosis [2].

#### S100 calcium‐binding protein B

S100B is abundant in glial cells expressed in astrocytes surrounding the blood vessels in the brain and in the Schwann cells of the peripheral nervous system, where they stimulate cellular processes such as proliferation, differentiation, and regulation of intracellular Ca^2+^ homeostasis. At least 80–90% of the total S100B pool is found within the brain, the remainder being located in other non-neuronal tissues. S100B is released from damaged astrocytes into the bloodstream after ischemic–reperfusion injury that occur after CA. Is considered an early biomarker, as the level usually peaks at 24 h and elevated levels are associated with poor outcome [35].

#### Neurofilament light

Neurofilament light chain (NfL) is a subunit of neurofilaments, which are cylindrical proteins exclusively located in the neuronal cytoplasm, predominantly within large, myelinated axons within the cerebral white matter. Their function is largely unknown but hypothesized to be essential for radial growth and enabling rapid nerve conduction [35]. Under physiological conditions, low levels of NfL are constantly released from axons, probably in an age-dependent manner, however, in response to CNS axonal damage because of pathological problem, such as an ischemic insult as occurs during CA, the release of NfL sharply increases. High levels in CA patients are index of poor outcomes [35].

#### Lactate

Lactate, a product of pyruvate reduction during glycolysis, has been suggested to be an indicator of multi-organ failure hypoxia resulting from reduced cardiac output and in CA patients elevated arterial blood lactate levels are associated with poor neurologic outcome [36]. Lactatemia during the first hours after CA is associated with hypoperfusion after the cessation of blood flow and the inflammatory reaction due to ischemia–reperfusion injury [36]. Hyperlactatemia few h after ROSC may indicate complication in patients, such as poor neurological function. Hence, lactate levels in CA are a critical marker for assessing the severity of ischemic–reperfusion injury, as well as predicting neurological outcomes.

### Electroencephalography

Electroencephalography (EEG) is a highly sensitive tool for detecting the severity of PCABI since assesses cortical synaptic activity [5]. Moreover, EEG is able to evaluate the occurrence of seizures as well as the appearance of spikes/sharp waves and epileptiform discharges. These, together with malignant EEG patterns (persistent iso-electricity, low voltage activity, or low burst-suppression patterns), are used to prognosticate outcome after CA. ERC guidelines 2021 suggest that highly malignant EEG after 24 h is an indicator of poor neurological outcome [2].

### Imaging

Brain CT is extensively used shortly after CA to rule out neurological causes of arrest, especially an intracranial hemorrhage that would contraindicate percutaneous coronary interventions. However, CT also allows assessing the severity of PCABI by detecting brain oedema [1].

The use of MRI for prognostication is more recent, but has rapidly gained interest during the last decade. One of the main advantages of MRI is the ability to assess the anatomical distribution of diffusion restrictions [37]. MRI allows the detection of cytotoxic edema, which occurs within hours after CA. Restricted diffusion by cytotoxic edema can be quantified by the Apparent Diffusion Coefficient (ADC) value of each voxel. Low ADC values, thus restricted water diffusion (DWI lesions) are associated with poor outcome after CA [2]. Moreover, an ADC values < 650X10^−6^ mm^2^/s in > 10% of the brain at 7 days after CA is highly specific for poor outcome.

DTI is an extension of DWI that allows the evaluation of microstructural integrity of brain white matter by directional assessment of water diffusion. Although DTI is not a criterion in the strategy algorithm for neuroprognostication, it was found that changes in DTI parameters can predict poor outcome in CA patients with 85% sensitivity [37].

### Bridging the gap between preclinical and clinical studies

Our review highlights several gaps between preclinical models and clinical practices. Preclinical studies often rely heavily on brain histopathology as a primary endpoint, neglecting correlations with functional outcomes or biomarkers. Furthermore, animal models typically involve young, healthy subjects, which do not adequately reflect the comorbidities present in clinical patients.

Significant correlations are seen between imaging, neurological deficits, and biomarkers, reflecting the severity of brain injury in CA patients, suggesting similar correlations should be explored in preclinical models. Indeed, in our analysis we did not find studies that explored possible correlations between outcomes. Interestingly, our review highlights a significant gap in the literature, as very few studies have explored potential correlations between histology, neuroimaging, electrophysiology, blood biomarkers, and neurobehavioral outcomes. Notably, there are only two studies addressing these correlations that demonstrated strong, positive correlations between apparent diffusion coefficient (ADC) values and the neurological deficit score, showing that the severity of brain cytotoxic edema is closely associated with worsened neurological function in the early phase after CA [8, 9]. In addition, one of these studies found a strong correlation between diffusion tensor imaging (DTI) metrics and the brain injury biomarker NfL [8]. These findings provide compelling evidence that such correlation can be identified underscoring a critical parallel between preclinical models and the clinical scenarios, emphasizing the translational relevance of incorporating multimodal assessments in preclinical research.

To improve translational value, preclinical models should integrate multimodal assessments and better mimic clinical conditions providing a more comprehensive assessment of the brain during CA.

## Conclusion

Our review advocates for the adoption of a multimodal approach in preclinical studies, integrating neuroimaging, biomarkers, and EEG, to better reflect the severity of brain injury, similar to the strategies used in clinical neuroprognostication. These approaches must always be integrated with histology, which plays a pivotal role in preclinical studies in understanding the pathophysiological mechanisms underlying PCABI. This comprehensive approach aligns with clinical methodologies, where the combination of multiple assessment tools provides a more reliable evaluation of neurological outcomes in patients. By implementing such a multimodal strategy in preclinical research, we can improve the translational relevance of experimental findings, helping to bridge the gap between laboratory models and clinical practice.

## Data Availability

The data sets generated and/or analyzed during the current study are available at: https://zenodo.org/records/13928458
